# Clone-specific expression, transcriptional regulation, and action of interleukin-6 in human colon carcinoma cells

**DOI:** 10.1186/1471-2407-8-13

**Published:** 2008-01-18

**Authors:** Wolfgang Brozek, Giovanna Bises, Gerhild Fabjani, Heide S Cross, Meinrad Peterlik

**Affiliations:** 1 Department of Pathophysiology, Medical University of Vienna, Vienna, Austria; 2 Department of Obstetrics and Gynaecology, Medical University of Vienna, Vienna, Austria; 3 Ludwig Boltzmann Institute for Gynaecological Oncology and Reproductive Medicine, Vienna, Austria

## Abstract

**Background:**

Many cancer cells produce interleukin-6 (IL-6), a cytokine that plays a role in growth stimulation, metastasis, and angiogenesis of secondary tumours in a variety of malignancies, including colorectal cancer. Effectiveness of IL-6 in this respect may depend on the quantity of basal and inducible IL-6 expressed as the tumour progresses through stages of malignancy. We therefore have evaluated the effect of *IL-6 *modulators, i.e. IL-1β, prostaglandin E_2_, 17β-estradiol, and 1,25-dihydroxyvitamin D_3_, on expression and synthesis of the cytokine at different stages of tumour progression.

**Methods:**

We utilized cultures of the human colon carcinoma cell clones Caco-2/AQ, COGA-1A and COGA-13, all of which expressed differentiation and proliferation markers typical of distinct stages of tumour progression. IL-6 mRNA and protein levels were assayed by RT-PCR and ELISA, respectively. DNA sequencing was utilized to detect polymorphisms in the *IL-6 *gene promoter.

**Results:**

*IL-6 *mRNA and protein concentrations were low in well and moderately differentiated Caco-2/AQ and COGA-1A cells, but were high in poorly differentiated COGA-13 cells. Addition of IL-1β (5 ng/ml) to a COGA-13 culture raised IL-6 production approximately thousandfold via a prostaglandin-independent mechanism. Addition of 17β-estradiol (10^-7 ^M) reduced basal IL-6 production by one-third, but IL-1β-inducible IL-6 was unaffected. Search for polymorphisms in the *IL-6 *promoter revealed the presence of a single haplotype, i.e., -597A/-572G/-174C, in COGA-13 cells, which is associated with a high degree of transcriptional activity of the *IL-6 *gene. IL-6 blocked differentiation only in Caco-2/AQ cells and stimulated mitosis through up-regulation of c-*myc *proto-oncogene expression. These effects were inhibited by 10^-8 ^M 1,25-dihydroxyvitamin D_3_.

**Conclusion:**

In human colon carcinoma cells derived from well and moderately differentiated tumours, IL-6 expression is low and only marginally affected, if at all, by PGE_2_, 1,25-dihydroxyvitamin D_3_, and 17β-estradiol. However, IL-6 is highly abundant in undifferentiated tumour cells and is effectively stimulated by IL-1β. In case of overexpression of an *IL-6 *gene variant with extreme sensitivity to IL-1β, massive release of the cytokine from undifferentiated tumour cells may accelerate progression towards malignancy by paracrine action on more differentiated tumour cells with a still functioning proliferative IL-6 signalling pathway.

## Background

Interleukin-6 (IL-6) is an immunomodulatory cytokine [1], which also plays a role in growth stimulation, metastasis, and angiogenesis in secondary tumours in a variety of malignancies [2], including colorectal cancer [3-7]. IL-6 can be released from tumour infiltrating leukocytes [8], but is produced to a large extent by tumour cells themselves: In human colon cancer, IL-6 expression parallels tumour progression, reaching a maximum in high grade cancerous lesions [5]. Only still differentiated colon carcinoma cells are responsive to the growth stimulatory action of IL-6 [5]. We therefore reasoned that IL-6, when released from rather undifferentiated colon carcinoma cells, may aid tumour progression by a paracrine-induced proliferation of still differentiated neoplastic cells [5]. In addition, IL-6 increases invasiveness of colon cancer cells [6] and likely promotes secondary tumour formation through its angiogenic potency. How effective IL-6 is in promoting progression and metastatic spread of colon cancer, depends not only on the extent of basal but, importantly, on the extent of inducible IL-6 expression at certain stages of tumour development.

IL-6 expression is highly inducible in different cell types by a variety of cytokines, particularly IL-1β [9-12], by prostaglandins [12,13], steroid hormones, such as estrogen [14] or 1,25-dihydroxyvitamin D_3 _[13,15]. Although these substances influence colon carcinoma cell growth, their ability to regulate *IL-6 *activity in this cell type has not yet been evaluated in detail. The present study was initiated to assess the potential of the aforementioned agents in modulating *IL-6 *transcriptional activity and protein synthesis at different stages of human colon cancer progression.

## Methods

### Human colon carcinoma primary cell clones and cell lines

From the Caco-2 cell line (ATCC HTB-37), which was originally derived from a well differentiated human colon adenocarcinoma, a number of homogenous and stable Caco-2 clones were established in several laboratories. For the present study, we used the clone Caco-2/AQ, which was derived from the Caco-2/15 clonal line [16] as described before [17]. Caco-2/AQ cells undergo spontaneous differentiation on transition into the post-confluent state, as indicated by a steep rise in differentiation markers, i.e. alkaline phosphatase activity (cf. Table 1).

**Table 1:** Characteristics of human colon carcinoma cell lines

	Caco-2/AQ	COGA-1A	COGA-13
Log phase doubling time (h)	24	15	48
Alkaline phosphatase (mU/μg cellular protein)	60.2 ± 9.1	2.9 ± 0.1	0.7 ± 0.2
*Protein*			
CK8	+++	+++	+
Vimentin	+	+	+++
Cyclin D1	+	++	+++
p27	+++	++	+/-
*mRNA expression*			
CYP24			
basal	+/-	++	+++
after 1,25-(OH)_2_D_3_^*a*^	+++	+++	+++
CYP27B1	+++	+/-	+/-
VDR	+++	+++	++
ER-α	+/-	+/-	+/-
ER-β	+	+	+

For details see [5,19,24]. Protein and mRNA expression in confluent cells were classified by visual inspection of Western blots or RT-PCR gels as: nil, -; low, +; medium, ++; high, +++.

^*a *^after treatment with 1,25-(OH)_2_D_3 _(cf. [19])

Primary colon adenocarcinoma cell clones COGA-1 and COGA-13 were isolated by Drs. Ernst Wagner and Alexandra Sinski at Boehringer Ingelheim Austria, Vienna, as described in detail elsewhere [18]. From the COGA-1 clonal cell line, which was derived from a Dukes' stage B, pT3, moderately differentiated (i.e. G2) carcinoma, a morphologically homogenous sub-clone, designated COGA-1A, was established and characterised with respect to growth behaviour and degree of differentiation, i.e., alkaline phosphatase activity (Table 1; cf. [5]).

COGA-13 cells, which were derived from a stage pT2 carcinoma graded G3, exhibit only weak alkaline phosphatase activity (Table 1). In addition to the epithelial cell marker cytokeratin 8 (CK8), COGA-13 cells express also a high level of vimentin [19]; this indicates that they had undergone epithelial-mesenchymal transition.

### Cell culture

Human colon cancer cells were routinely cultured in vented tissue culture flasks (Asahi Techno Glass Corporation, Iwaki Scitech division, Tokyo, Japan) at 37°C in a humidified atmosphere of 95% air and 5% CO_2_. Culture medium was DMEM supplemented with 4.0 mM glutamine, 10% (v/v) fetal calf serum (FCS) (heat-inactivated at 56°C for 30 min), 20 mM HEPES, 50 U/ml penicillin and 50 μg/ml streptomycin. Cultures were re-fed every 48 h and subcultured serially when approximately 80% confluent. Cells between passages 6 and 24 were cultured for indicated time periods in the absence or presence of one of the following treatments: 5 ng/ml IL-1β (ImmunoTools, Friesoythe, Germany); 10^-6 ^M indomethacin, 10^-7 ^M PGE_2_, 10^-7 ^M 17β-estradiol, 10^-6 ^M NS398 (all from Sigma), and 10^-8 ^M 1,25-(OH)_2_D_3 _(a generous gift from Hoffmann-La Roche, Basle, Switzerland).

The effect of 0.1 – 100 ng/ml recombinant human (rh) IL-6 (Strathmann Biotec AG, Hamburg, Germany) on cellular proliferation was determined by measurement of [^3^H]thymidine incorporation into cellular DNA. Degree of cellular differentiation was evaluated in confluent cells from activity of the marker enzyme alkaline phosphatase as described in detail previously [20]: Enzymatic activity was assayed using *p*-nitrophenol as substrate, and normalised to cellular protein content, which was determined using the BCA Protein Assay Kit (Pierce, Rockford, IL).

### Reverse transcriptase polymerase chain reaction (RT-PCR)

Total RNA was extracted by using Trizol (GibcoBRL). 2 μg RNA were reverse transcribed with random hexamer primers using a cDNA synthesis kit (SuperScript™ II, Invitrogen). First-strand cDNA was amplified with primer pairs for IL-6, and for the reference gene and epithelial cell marker CK8, respectively (MWG-Biotech AG, Ebersberg, Germany). To amplify a 349 base pair segment of IL-6 cDNA, primer pairs used were 5'-TTC-AAT-GAG-GAG-ACT-TGC-CTG-3' (sense) and 5'-ACA-ACA-ACA-ATC-TGA-GGT-GCC-3' (antisense) [21]. The primer pairs for c-myc were 5'-GGC-TTT-ATC-TAA-CTC-GCT-GT-3' (sense) and 5'-GAG-GTC-ATA-GTT-CCT-GTT-GG-3' (antisense) to amplify a 461 base pair segment [22]. Primer pairs for a 520 base pair segment of the CK8 gene were 5'-TGG-GCA-GCA-GCA-TTA-ACT-TTC-3' (sense) and 5'-AGG-CGA-GAC-TCC-AGC-TCT-AC-3' (antisense) [23]. With the aid of the GeneAmp PCR System 9600 (Perkin-Elmer, Norwalk, CT), the thermocycling conditions began with 94°C for 2 min followed by 34 cycles (IL-6) and 30 cycles (CK8), respectively, of: 94°C 15 sec, 62°C 30 sec, 72°C 45 sec, or followed by 30 cycles (c-myc) of 94°C 30 sec, 57°C 60 sec, 72°C 60 sec. Each run was concluded by 10 min at 72°C. PCR conditions and primer pairs for cDNA amplification of CYP27B1, CYP24, and VDR were previously provided elsewhere [19], as well as for cDNA amplification of ER-α and ER-β [24]. A 1:1 mixture of PCR products and CK8 amplificates from the same sample was separated on 2% agarose gels and then visualised with ethidium bromide. No bands were visible in negative controls that were performed for all primer pairs.

### Determination of protein

IL-6 concentrations in cell culture supernatants were determined by enzyme-linked immunosorbent assay (ELISA) (eBioscience, San Diego, CA) according to the protocol of the manufacturer. Since preliminary data had indicated that some clones might produce IL-6 in amounts close to the detection limit of the assay (2 pg/ml IL-6), we added a constant background of 10 pg/ml IL-6 to all samples to improve intra-assay accuracy. Immunoblotting for CK8, vimentin, cyclin D1, and p27 has been described elsewhere [19].

### DNA isolation and sequence analysis

Human genomic DNA was extracted from confluent colorectal cell clones. Cells were trypsinised and washed twice with PBS before suspension in extraction buffer (50 mM Tris / HCl pH 8, 100 mM EDTA, 100 mM NaCl, 1% SDS, 0.5 mg/ml proteinase K) and overnight shaking on an Eppendorf shaker at 55°C. Saturated NaCl (6 M) (a third of the volume of extraction buffer) was added before centrifugation at 13000 rpm for 10 min on a Microfuge Lite Centrifuge (Beckman Coulter, Fullerton, CA). The supernatant was treated with approximately the same amount of isopropanol, shaken thoroughly, and centrifuged (13000 rpm, 5 min). Thereafter, the pellet was washed with EtOH (70%, v/v), resuspended in 400 μl TE buffer (10 mM Tris, pH 8 / 1 mM EDTA), and agitated on an Eppendorf shaker at 65°C for 15 min. DNA was stored at -20°C until analysed. DNA amplification was performed with a GeneAmp PCR System 9600 (Perkin-Elmer, Foster City, CA) as described previously [25]. PCR products were subsequently purified using the Centri Sep 96 system (Princeton Separations, NJ), and thereafter subjected to sequencing using Big Dye Terminator Cycling Sequencing kit, version v3.1, and the ABI Prism 310 Genetic Analyzer (Applied Biosystems, Foster City, CA) according to the manufacturer's recommendation. Primers for amplification and sequencing were applied as listed in [9].

### Statistical analysis

Statistical analyses were conducted with use of the software packages S-PLUS (version 4.5, Lucent Technologies Inc., Murray Hill, NJ) and SPSS (version 12.0.1, SPSS Inc., Chicago, IL). The normal distribution of all data sets was verified with the aid of the one-sample Kolmogorov-Smirnov goodness of fit-test; as a result the unpaired Student's *t*-test was used throughout. With a confidence level of 0.95, differences were considered statistically significant with *, *P *< 0.05; **, *P *< 0.01; ***, *P *< 0.001.

## Results

### Validation of human colon carcinoma cell clone models

We had availed ourselves of three human colon carcinoma-derived cell clones, i.e. Caco-2, COGA-1A, and COGA-13, which we had characterised previously with respect to their log phase growth behaviour and to expression of the differentiation marker alkaline phosphatase [5] (Table 1). For the purpose of the present study, the proliferative potential was evaluated by determination of the cell cycle regulators, cyclin D1, which promotes G1/S transition, and p27, a cdk-inhibitor. We also determined expression of the epithelial and mesenchymal cell markers, cytokeratin 8 (CK8) and vimentin, respectively. Furthermore, we checked for expression of the vitamin D metabolising enzymes, 25-hydroxy-vitamin D-24-hydroxylase (CYP24) and 25-hydroxy-vitamin D-1α-hydroxylase (CYP27B1), as well as of the vitamin D receptor (VDR), and the estrogen receptors (ER)-alpha and -beta (Table 1).

Consistent with their origin from a well differentiated tumour, Caco-2 cells exhibited the highest level of the differentiation marker alkaline phosphatase. In contrast, enzyme activity was low in COGA-1, and was barely detectable in COGA-13 cells. Cellular differentiation declined in the order Caco-2 > COGA-1A > COGA-13. The proliferative potential varied inversely with differentiation, as indicated by the rise in cyclin D1 and the drop in p27 (Table 1).

All three cell clones were of epithelial origin according to their expression of the epithelial cell marker CK8 (Table 1). Little vimentin was detected in Caco-2 and COGA-1A cells, whereas in COGA-13, expression of vimentin exceeded that of CK8. This indicates a high degree of epithelial/mesenchymal transition in these least differentiated cancer cells (Table 1).

The findings listed in Table 1 also indicate that all three clones, notwithstanding marked differences in growth characteristics and degree of differentiation, express the VDR, and, as typical for colon epithelial cells, the ER-β [24]. Consequently, all three clones can be expected to respond to the receptor ligands, i.e., 1,25-(OH)_2_D_3 _and 17β-E_2_. Also, all three clones expressed the vitamin D metabolising enzymes, 25-hydoxyvitamin D-24-hydroxylase (CYP24) and 25-hydoxyvitamin D-1α-hydroxylase (CYP27B1), though at markedly different levels. Of note, CYP27B1 was abundant in Caco-2/AQ cells, as is typical for rather differentiated colon carcinomas, whereas CYP24 was overexpressed in COGA-13 cells consistent with a low degree of differentiation [26].

### IL-6 mRNA and protein expression in human colon carcinoma cells: effects of IL-1β, PGE_2_, 1,25-(OH)_2_D_3_, and 17β-E_2_

**Figure 1:**
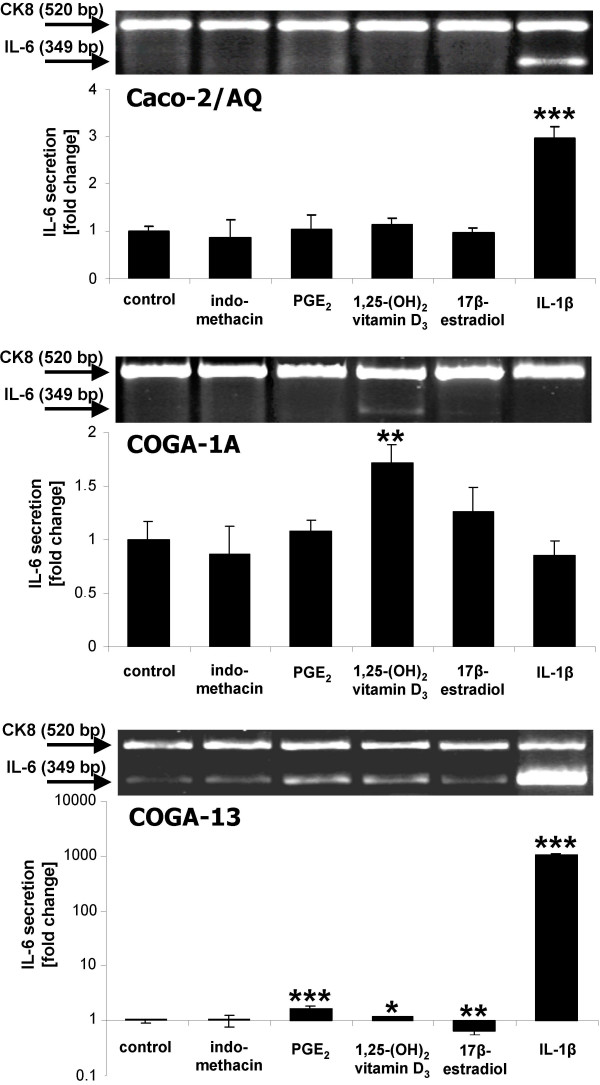
Basal and stimulated expression of IL-6 mRNA and protein in human colon carcinoma cell clones. Additions to the culture medium were: 10^-6 ^M indomethacin, 10^-7 ^M PGE_2_, 10^-8 ^M 1,25-(OH)_2_D_3_, 10^-7 ^M 17β-E_2_, 5 ng/ml IL-1β. Upper part: Representative RT-PCR amplifications of mRNA transcripts specific for *IL-6 *(culture time 4 h). Expression of epithelial cell marker CK8 is shown for comparison. Lower part: IL-6 release into medium during 24 h culture period. Data are means ± SD, *n *≥ 4. Note that changes in IL-6 secretion by COGA-13 cells are given on a logarithmic scale. Statistically significant differences: *, *P *< 0.05; **, *P *< 0.01; ***, *P *< 0.001 (Student's *t*-test).

RT-PCR analysis revealed a distinct, i.e., clone-specific, pattern of basal and stimulated expression of IL-6 mRNA in human colon carcinoma cells (Figure 1): As had been observed previously, carcinoma cell clones derived from well to moderately differentiated cancers, i.e. Caco-2 and COGA-1A, expressed only small amounts of IL-6, as compared with COGA-13 cells, which are derived from a poorly differentiated tumour. In these cells, which had already undergone substantial epithelial-mesenchymal transition, IL-6 is conspicuously overexpressed.

In Caco-2/AQ cells, IL-6 gene activity and protein synthesis were up-regulated only by IL-1β, whereas COGA-1A responded only to 1,25-(OH)_2_D_3_. In COGA-13 cells, IL-6 levels were increased to some extent by PGE_2_, whereas IL-1β increased IL-6 expression by three orders of magnitude (Figure 1).

A more detailed picture of clonal sensitivity towards modulators of IL-6 was obtained when IL-1β, PGE_2_, 1,25-(OH)_2_D_3_, and 17β-E_2_, singly or in appropriate combinations, were added to colon carcinoma cell cultures (Table 2). Overexpression of *IL-6 *by the COGA-13 clone became apparent also at the protein level, as COGA-13 cells produced at least four times more IL-6 than Caco-2/AQ or COGA-1A cells (Table 2). In none of the clones, IL-6 production, regardless of whether basal or IL-1β-induced, was changed when endogenous prostaglandin synthesis was blocked by indomethacin (Table 2) or by the cyclooxygenase (COX)-2 inhibitor, NS398 (not shown). As mentioned before, when 10^-7 ^M PGE_2 _was added to COGA-13 cultures, a small increment in IL-6 release was observed.

**Table 2:** Effect of modulators of IL-6 synthesis in human colon carcinoma cell clones

	IL-6 secretion by human colon carcinoma cell clones					
Addition	Caco-2/AQ		COGA-1A		COGA-13	

	pg/ml	fold change	pg/ml	fold change	pg/ml	fold change
			
None	4.86 ± 0.49	1.00 ± 0.10	5.49 ± 0.96	1.00 ± 0.17	21.20 ± 1.56	1.00 ± 0.07
indomethacin		0.88 ± 0.36		0.87 ± 0.25		1.02 ± 0.27
PGE_2_		1.03 ± 0.30		1.08 ± 0.11		1.79 ± 0.20***
1,25-(OH)_2_D_3_		1.13 ± 0.14		1.72 ± 0.17**		1.20 ± 0.03*
17β-E_2_		0.98 ± 0.08		1.26 ± 0.24		0.67 ± 0.12**
IL-1β		2.96 ± 0.26***		0.85 ± 0.13		1035 ± 73***
IL-1β *plus *indomethacin		3.12 ± 0.28***		0.93 ± 0.28		1147 ± 197***
IL-1β *plus *PGE_2_		2.83 ± 0.42***		0.92 ± 0.29		1366 ± 210***
IL-1β *plus *17β-E_2_		2.62 ± 0.24***		0.79 ± 0.12*		1071 ± 33***

Additions to culture medium for 24 h were: indomethacin, 10^-6 ^M; PGE_2_, 10^-7 ^M; 1,25-(OH)_2_D_3_, 10^-8 ^M; 17β-E_2_, 10^-7 ^M; IL-1β, 5 ng/ml.

17β-E_2 _was effective in COGA-13 cultures, where it reduced basal IL-6 release by one third. However, the hormone did not alter the extensive stimulation by IL-1β of IL-6 synthesis (Table 2).

### Sequence analysis of IL-6 promoter

We analysed the *IL-6 *gene promoter for the presence of specific polymorphisms that are known to influence transcriptional regulation of *IL-6*, e.g., by IL-1 and PGE_2 _[9] (Table 3).

**Table 3:** Genotypes at three common polymorphic sites of the *IL-6 *promoter in human colon carcinoma cell clones

Cell clones	Polymorphisms		
	-597G→A	-572G→C	-174G→C
Caco-2/AQ	G/A	G/G	G/C
COGA-1A	G/G	G/G	G/G
COGA-13	A/A	G/G	C/C

### IL-6 effect on growth and differentiation of human colon carcinoma cell clones

**Figure 2:**
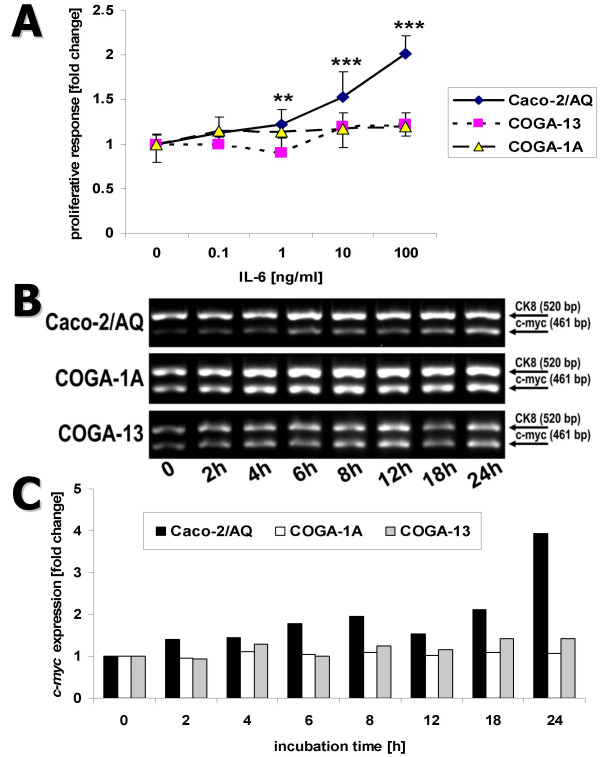
(A) Effect of hIL-6 on growth rate of confluent Caco-2/AQ, COGA-1A, and COGA-13 cells. Cellular proliferation was evaluated from [^3^H]thymidine incorporation into DNA after 72 h incubation with rhIL-6 (0–100 ng/ml). Data are means ± SD (*n *= 4 – 16) and expressed as multiples of IL-6-free controls. Statistically significant differences from controls: **, *P *< 0.01; ***, *P *< 0.001 (Student's *t*-test). (B) Time-course of *c-myc *mRNA expression in confluent Caco-2/AQ, COGA-1A, and COGA-13 clones during incubation with 100 ng/ml rhIL-6. Expression of the epithelial cell marker CK8 is shown for comparison. (C) Densitometric evaluation of expression of *c-myc *in relation to CK8 as shown in (B): basal expression ratios in zero time controls were set to 1.

To evaluate the mitogenic potency of IL-6 in Caco-2/AQ, COGA-1A and COGA-13 cell cultures, graded concentrations of the cytokine (0–100 ng/ml) were added to the medium for 72 h. A dose dependent increase in cell division was observed only in Caco-2/AQ cells, whereas the growth rate in COGA-1A and GOGA-13 was unaltered (Figure 2A). All three clones are endowed with the IL-6 receptor [5], but IL-6 proliferative signaling was transduced to *c-my*c expression only in Caco-2 cells (Figure 2B): IL-6 expression rose about fourfold after 24 h incubation with 100 ng/ml rhIL-6 (Figure 2C).

**Figure 3:**
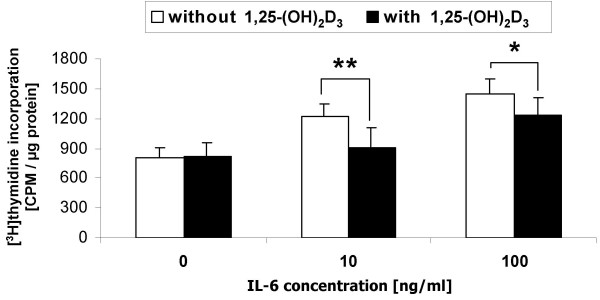
Effect of 1,25-(OH)_2_D_3 _(10^-8 ^M) on IL-6-related proliferation of confluent Caco-2/AQ cells. Culture time was 72 h. Cell growth was assayed by [^3^H]thymidine incorporation into cellular DNA (normalised to total protein). Data are expressed as means ± SD, *n *= 4 – 7. Statistically significant differences from controls: *, *P *< 0.05; **, *P *< 0.01 (Student's *t*-test).

**Figure 4:**
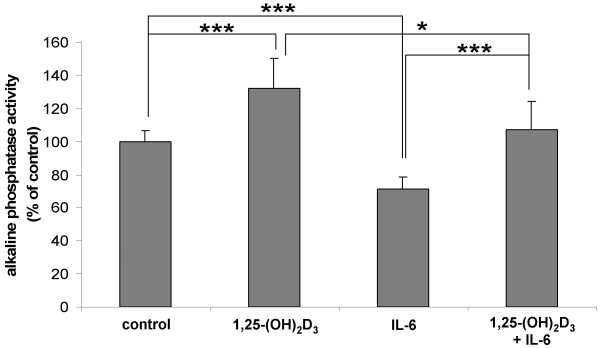
Effect of 1,25-(OH)_2_D_3 _on rhIL-6-induced inhibition of differentiation of Caco-2/AQ cells. Incubation time was 72 h. Concentrations in culture medium: 1,25-(OH)_2_D_3_, 10^-8 ^M; rhIL-6, 100 ng/ml. Cellular differentiation was evaluated from activity of the marker enzyme alkaline phosphatase. Data were calculated as mU/μg cellular protein and expressed as "percent of control" (means ± SD, *n *= 8). Statistically significant differences: *, *P *< 0.05; ***, *P *< 0.001 (Student's *t*-test).

In Caco-2 cell cultures, 10^-8 ^M 1,25-(OH)_2_D_3 _abolished the growth stimulatory effect of 10 ng/ml IL-6. Still a 50% reduction was observed when cell division was stimulated by IL-6 at the supraphysiological concentration of 100 ng/ml (Figure 3). 1,25-(OH)_2_D_3 _completely prevented any inhibitory effect of IL-6 on the activity of the differentiation marker enzyme, alkaline phosphatase (Figure 4).

## Discussion

The purpose of the present study was to evaluate the regulation of *IL-6 *activity in human colon carcinoma cells by classical modulators in respect to possible consequences for tumour progression. In this regard, it is important to note that each of the cell clones used in the present study, i.e., Caco-2/AQ, COGA-1A, and COGA-13, according to the characteristics shown in Table 1, represents a valid model system for studies on differentiation-related changes of human colon cancer cell functions during tumour progression.

Overexpression of *IL-6 *seems to be a hallmark of advanced tumour progression, since it has been observed, apart from colon cancer [5], also in other human malignancies, e. g., multiple myeloma, Kaposi's sarcoma [9], or glioblastoma [27]. From data reported in Figure 1 it is clear that human colon carcinoma cell clones derived from well to moderately differentiated tumours, i.e. Caco-2/AQ and COGA-1A, express relatively little IL-6, particularly when compared to the rather undifferentiated clone COGA-13. Not only *IL-6 *gene activity differs according to the degree of differentiation, but also the extent of unstimulated translation into protein is at least four times higher in COGA-13 than in Caco-2 or COGA-1A (Table 2).

The promoter region of the *IL-6 *gene contains a number of binding sites for transcription factors such as NFIL6 (C/EBPβ), NFκB, Fos/Jun (AP-1), CRBP, CREB etc. [9-12]. This explains the basic sensitivity of *IL-6 *towards classical transcription modulating factors such as prostaglandins, cytokines, steroid hormones etc. It is interesting to note that PGE_2 _at 10^-7 ^M had no effect on *IL-6 *expression and protein synthesis in Caco-2 and COGA-1A cells, and induced only a relatively small change in COGA-13 cells (Table 2). Insensitivity of *IL-6 *to PGE_2 _can be deduced also from the observation that in all three cell clones, though they are endowed with cyclooxygenase-2 (COX-2) activity [28], suppression of endogenous PG synthesis by indomethacin or NS398 had no effect on basal and IL-1β-stimulated *IL-6 *expression and protein secretion (Table 2). We conclude from this that in human colon cancer cells, endogenous PG production, even when stimulated by IL-1β [28], is too low to affect IL-6 production, and that, conversely, the chemopreventive effect on colon cancer development of COX-2 inhibitors [29] does not involve changes of *IL-6 *expression.

Although all cell types investigated had been shown to express ER-α and -β as well as the VDR (cf. Table 1), *IL-6 *expression in human colon carcinoma cells was only modestly, if at all, influenced by the steroid hormones 17β-E_2 _and 1,25-(OH)_2_D_3 _(Figure 1; Table 2). 17β-E_2 _had no effect on IL-6 synthesis in Caco-2/AQ and COGA-1A cells, and inhibited IL-6 production by highly undifferentiated COGA-13 cells only to an extent which makes efficient suppression of IL-6 production in high grade cancers by oestrogens very unlikely. This implies that prevention of human colorectal cancer by oestrogens [30] does not involve any direct effect on *IL-6 *expression.

Resistance to 1,25-(OH)_2_D_3 _in VDR-positive cells can be due to rapid degradation of the steroid catalysed by the 25-(OH)-D-24-hydroxylase (cf. Table 1). This could explain why 1,25-(OH)_2_D_3 _has no effect on IL-6 production in COGA-13, or only a marginal one in COGA-1A cells, but this is certainly not valid for Caco-2 cells, which are generally responsive to VDR-mediated actions of 1,25-(OH)_2_D_3 _[19]. The small 1,25-(OH)_2_D_3_-related increment of IL-6 production by COGA-1A cells seems to be without relevance for the anti-mitogenic and pro-differentiating effects of the hormone in human colon carcinoma cells [19], since 1,25-(OH)_2_D_3 _effectively suppressed IL-6-induced growth in differentiated Caco-2 cells (Figure 3). At the same time, the pro-differentiating action of 1,25-(OH)_2_D_3 _was completely preserved, even at extremely high IL-6 concentrations (Figure 4).

The inefficiency of 17β-E_2 _and 1,25-(OH)_2_D_3 _in modulating *IL-6 *transcriptional activity could result from impaired or abrogated signal transduction downstream of the ER-β or VDR, respectively, but may also be due to clone-specific expression of *IL-6 *gene variants. This could be the consequence of acquisition of mutations during tumour development and progression, or, respectively, caused by specific polymorphisms in the *IL-6 *promoter. Terry *et al*. [9] had identified four polymorphic sites, which influence not only basal but also regulatable transcription in a complex cooperative manner [12]. For example, the -174C *IL-6 *haplotype is less efficiently translated into protein than its -174G counterpart, and conveys resistance of *IL-6 *to the stimulatory action of IL-1 [12]. This may be the reason why Belluco *et al*. [31] found that colon cancer patients carrying the -174G polymorphic *IL-6 *gene had significantly higher IL-6 serum levels than patients with the -174C genotype, particularly in the presence of hepatic metastases.

Our search for promoter polymorphisms (Table 3) showed that all cell clones investigated express *IL-6 *variants which were identical only at -572 but different from each other at sites -597 and -174. The -597A/-572G/-174C haplotype, as solely present in COGA-13 cells, has been identified by Terry *et al*. [9] as the one which, when transfected in ECV304 cells, shows a comparable high transcriptional activity (cf. also [12]). This may explain why COGA-13 cells, particularly since they overexpress *IL-6*, produce significantly more IL-6 than Caco-2 or COGA-1A cells (Table 2). However, the same -597A/-572G/-174C haplotype shows the least sensitivity to IL-1β [9]. Therefore, the striking difference in the regulation of transcriptional activity of *IL-6 *by IL-1β (cf. Table 2) could only be due to cooperativity with still unknown promoter polymorphisms or, much more likely, due to mutational changes in the *IL-6 *gene acquired during progression through the adenoma/carcinoma sequence. These questions can only be answered when more information on polymorphic sites and cancer-related mutations in the IL-6 gene will be available.

From the results of the present study it is conceivable that a highly critical situation for colon cancer patients may arise, when COGA-13-type cells become abundant in a cancerous lesion. If unopposed, massive release of IL-6 under the stimulation by IL-1β might accelerate tumour progression to high stage malignancy by paracrine proliferative action on IL-6-responsive, i.e., still differentiated cells. At present, we are unaware of any means by which IL-6 secretion from undifferentiated colon cancer cells can be effectively suppressed. Alternatively, development of anti-IL-6 or anti-IL-6 receptor monoclonal antibodies could be beneficial for future adjuvant immune therapy for cancer patients with genetic predisposition for IL-6 overexpression. In any case, screening for carriers of IL-6 gene variants with high susceptibility to transcriptional dysregulation by IL-1β should be considered for identification of individuals with high-risk for therapy-resistant colorectal cancer.

## Conclusion

Evidence is provided that in human colon cancer, undifferentiated tumour cells are the main source of IL-6. The cytokine can be released in massive amounts, particularly when its expression is up-regulated by IL-1β. Transduction of IL-6 signalling into up-regulation of *c-myc *expression results in enhanced growth of colon carcinoma cells.

## Competing interests

The authors declare that they have no competing interests.

## Authors' contributions

WB carried out the proliferation assays, RT-PCR and protein analyses, and performed the statistical analyses, GB helped with the cell culture experiments for characterisation of the colon carcinoma cell clones, GF carried out the analyses of polymorphisms in the *IL-6 *gene promoter, HSC participated in the design of the study and coordinated all the experimental work, MP conceived of the study, participated in its design and drafted the manuscript. All authors read and approved the final manuscript.

## Pre-publication history

The pre-publication history for this paper can be accessed here:



## References

[B1] Hirano T (1998). Interleukin 6 and its receptor: ten years later. Int Rev Immunol.

[B2] Ardestani SK, Inserra P, Solkoff D, Watson RR (1999). The role of cytokines and chemokines on tumor progression: a review. Cancer Detect Prev.

[B3] Shirota K, LeDuy L, Yuan S, Jothy S (1990). Interleukin-6 and its receptor are expressed in human intestinal epithelial cells. Virchows Arch B.

[B4] Schneider MR, Hoeflich A, Fischer JR, Wolf E, Sordat B, Lahm H (2000). Interleukin-6 stimulates clonogenic growth of primary and metastatic human colon carcinoma cells. Cancer Lett.

[B5] Brozek W, Bises G, Girsch T, Cross HS, Kaiser HE, Peterlik M (2005). Differentiation-dependent expression and mitogenic action of interleukin-6 in human colon carcinoma cells: Relevance for tumour progression. Eur J Cancer.

[B6] Hsu C-P, Chung Y-C (2006). Influence of interleukin-6 on the invasiveness of human colorectal carcinoma. Anticancer Res.

[B7] Street ME, Miraki-Moud F, Sanderson IR, Savage MO, Giovanelli G, Bernasconi S, Camacho-Hübner C (2003). Interleukin-1β (IL-1β) and IL-6 modulate insulin-like growth factor-binding protein (IGFBP) secretion in colon cancer epithelial (Caco-2) cells. J Endocrinol.

[B8] Atreya R, Neurath MF (2005). Involvement of IL-6 in the pathogenesis of inflammatory bowel disease and colon cancer. Clin Rev Allergy Immunol.

[B9] Terry CF, Loukaci V, Green FR (2000). Cooperative influence of genetic polymorphisms on interleukin 6 transcriptional regulation. J Biol Chem.

[B10] Ray A, Tatter SB, May LT, Sehgal PB (1988). Activation of the human "*β*_2_-interferon/hepatocyte-stimulating factor/interleukin 6" promoter by cytokines, viruses, and second messenger agonists. Proc Natl Acad Sci USA.

[B11] Isshiki H, Akira S, Tanabe O, Nakajima T, Shimamoto T, Hirano T, Kishimoto T (1990). Constitutive and interleukin-1 (IL-1)-inducible factors interact with the IL-1-responsive element in the IL-6 gene. Mol Cell Biol.

[B12] Fishman D, Faulds G, Jeffery R, Mohamed-Ali V, Yudkin JS, Humphries S, Woo P (1998). The effect of novel polymorphisms in the interleukin-6 (IL-6) gene on IL-6 transcription and plasma IL-6 levels, and an association with systemic-onset juvenile chronic arthritis. J Clin Invest.

[B13] Gruber R, Nothegger G, Ho G-M, Willheim M, Peterlik M (2000). Differential stimulation by PGE_2 _and calcemic hormones of IL-6 in stromal/osteoblastic cells. Biochem Biophys Res Comm.

[B14] Schiller C, Gruber R, Redlich K, Ho G-M, Katzgraber F, Willheim M, Pietschmann P, Peterlik M (1997). 17*β*-Estradiol antagonizes effects of 1*α*,25-dihydroxyvitamin D_3 _on interleukin-6 production and osteoclast-like cell formation in mouse bone marrow primary cultures. Endocrinology.

[B15] Willheim M, Thien R, Schrattbauer K, Bajna E, Holub M, Gruber R, Baier K, Pietschmann P, Reinisch W, Scheiner O, Peterlik M (1999). Regulatory effects of 1*α*, 25-dihydroxyvitamin D_3 _on the cytokine production of human peripheral blood lymphocytes. J Clin Endocrinol Metab.

[B16] Beaulieu J-F, Quaroni A (1991). Clonal analysis of sucrase-isomaltase expression in the human colon adenocarcinoma Caco-2 cells. Biochem J.

[B17] Hofer H, Ho G-M, Peterlik M, Uskokoviæ MR, Lee J-K, White MC, Posner GH, Cross HS (1999). Biological effects of 1*α*-hydroxy- and 1*β*-(hydroxymethyl)-vitamin D compounds relevant for potential colorectal cancer therapy. J Pharmacol Exp Ther.

[B18] Vécsey-Semjén B, Becker KF, Sinski A, Blennow E, Vietor I, Zatloukal K, Beug H, Wagner E, Huber LA (2002). Novel colon cancer cell lines leading to better understanding of the diversity of respective primary cancers. Oncogene.

[B19] Lechner D, Kállay E, Cross HS (2007). 1α,25-Dihydroxyvitamin D_3 _downregulates CYP27B1 and induces CYP24A1 in colon cells. Mol Cell Endocrinol.

[B20] Zucco F, Batto AF, Bises G, Chambaz J, Chiusolo A, Consalvo R, Cross H, Dal Negro G, de Angelis I, Fabre G, Guillou F, Hoffman S, Laplanche L, Pinçon-Raymond M, Prieto P, Turco L, Ranaldi G, Rousset M, Sambuy Y, Scarino ML, Torreilles F, Stammati A (2005). An inter-laboratory study to evaluate the effects of medium composition on the differentiation and barrier function of Caco-2 cell lines. Altern Lab Anim.

[B21] Faruqi TR, Gomez D, Bustelo XR, Bar-Sagi D, Reich NC (2001). Rac1 mediates STAT3 activation by autocrine IL-6. Proc Natl Acad Sci USA.

[B22] Hashimoto K, Nakagawa Y, Morikawa H, Niki M, Egashira Y, Hirata I, Katsu K, Akao Y (2001). Co-overexpression of DEAD box protein rck/p54 and c-myc protein in human colorectal adenomas and the relevance of their expression in cultured cell lines. Carcinogenesis.

[B23] Shiratsuchi H, Saito T, Sakamoto A, Itakura E, Tamiya S, Oshiro Y, Oda Y, Toh S, Komiyama S, Tsuneyoshi M (2002). Mutation analysis of human cytokeratin 8 gene in malignant rhabdoid tumor: a possible association with intracytoplasmic inclusion body formation. Mod Pathol.

[B24] Lechner D, Bajna E, Adlercreutz H, Cross HS (2006). Genistein and 17β-estradiol, but not equol, regulate vitamin D synthesis in human colon and breast cancer cells. Anticancer Res.

[B25] Wieser F, Fabjani G, Tempfer C, Schneeberger C, Sator M, Huber J, Wenzl R (2003). Analysis of an interleukin-6 gene promoter polymorphism in women with endometriosis by pyrosequencing. J Soc Gynecol Investig.

[B26] Bareis P, Bises G, Bischof MG, Cross HS, Peterlik M (2001). 25-Hydroxy-vitamin D metabolism in human colon cancer cells during tumor progression. Biochem Biophys Res Comm.

[B27] Tchirkov A, Khalil T, Chautard E, Mokhtari K, Véronèse L, Irthum B, Vago P, Kémény J-L, Verrelle P (2007). Interleukin-6 gene amplification and shortened survival in glioblastoma patients. Br J Cancer.

[B28] Duque J, Díaz-Muñoz MD, Fresno M, Iñiguez MA (2006). Up-regulation of cyclooxygenase-2 by interleukin-1β in colon carcinoma cells. Cell Signal.

[B29] Fosslien E (2000). Molecular pathology of cyclooxygenase-2 in neoplasia. Ann Clin Lab Sci.

[B30] Rossouw JE, Anderson GL, Prentice RL, LaCroix AZ, Kooperberg C, Stefanick ML, Jackson RD, Beresford SAA, Howard BV, Johnson KC, Kotchen JM, Ockene J (2002). Risks and benefits of estrogen plus progestin in healthy postmenopausal women: principal results from the Women's Health Initiative randomized controlled trial. JAMA.

[B31] Belluco C, Olivieri F, Bonafè M, Giovagnetti S, Mammano E, Scalerta R, Ambrosi A, Franceschi C, Nitti D, Lise M (2003). *-174 G>C *polymorphism of *interleukin 6 *gene promoter affects interleukin 6 serum level in patients with colorectal cancer. Clin Cancer Res.

